# Draft Genome Sequence of High-Temperature-Adapted Protochlamydia sp. HS-T3, an Amoebal Endosymbiotic Bacterium Found in Acanthamoeba Isolated from a Hot Spring in Japan

**DOI:** 10.1128/genomeA.01507-14

**Published:** 2015-02-05

**Authors:** Hiroyuki Yamaguchi, Junji Matsuo, Tomohiro Yamazaki, Kasumi Ishida, Kenji Yagita

**Affiliations:** a Department of Medical Laboratory Science, Faculty of Health Sciences, Hokkaido University, Kita-ku, Sapporo, Japan; b Japan Society for the Promotion of Science, Chiyoda-ku, Tokyo, Japan; c National Institute of Infectious Diseases, Shinjuku-ku, Toyama, Tokyo, Japan

## Abstract

Here, we report the draft genome sequence of high-temperature-adapted Protochlamydia sp. strain HS-T3, an environmental chlamydia. This bacterium is an amoebal endosymbiont, found in Acanthamoeba isolated from a hot spring in Japan. Strain HS-T3 readily grew in mammalian cells at 37°C, a characteristic not previously reported for environmental chlamydiae.

## GENOME ANNOUNCEMENT

Chlamydiae is a bacterial phylum and class comprising obligate intracellular bacteria. Two distinct lineages exist, pathogenic and environmental chlamydiae, and this divergence likely occurred 0.7 to 1.4 billion years ago (1). Pathogenic chlamydiae, including Chlamydia trachomatis and Chlamydia pneumoniae, are well-known human pathogens causing ocular infection resulting in trachoma (preventable blindness) (2), sexually transmitted diseases with an estimated five million new cases annually worldwide (3), and community-acquired pneumonia (4). However, evidence showing an association between environmental chlamydiae and pathogenesis is limited. Pathogenic chlamydiae have successfully adapted to growth in mammalian cells, including human cells (which provide a stable environment with minimal temperature changes) through genome reduction, with an average genomic size of around 1.0 Mb (5). In contrast, environmental chlamydiae have co-evolved with their invertebrate hosts as endosymbionts of lower eukaryotes, free-living amoebae such as Acanthamoeba, adapting to a less hospitable environment with a relatively low temperature, likely without genome reduction (1, 6). Most environmental chlamydiae therefore have not successfully been cultured in mammalian cells at the general culture temperature of 37°C (7, 8). However, we recently identified an environmental chlamydia, high-temperature-adapted Protochlamydia sp. strain HS-T3, which can readily grow in human immortal HEp-2 epithelial cells under normal culture conditions. This strain was identified in Acanthamoeba isolated from a hot spring in Kanagawa prefecture, Japan (9).

The draft genome of Protochlamydia HS-T3 was obtained using an Illumina HiSeq 2000 sequencer (Illumina, San Diego, CA, USA), with sequencing runs for paired-end sequences. The bacterial DNA libraries were prepared using a TruSeq DNA sample kit (Illumina). The genome was assembled into 34 contigs, ranging in size from 189 to 363,061 bp, using *de novo* sequence assembler software (Velvet, EMBL-EBI) (10). Gene prediction was performed with Rapid Annotation using Subsystem Technology (RAST) (http://rast.nmpdr.org/) (11), and functional annotation was performed using the Kyoto Encyclopedia of Genes and Genomes (KEGG) (http://www.genome.jp/kegg/) (12). The sequencing runs and read assembly of the libraries were carried out by Hokkaido System Science (Sapporo, Japan).

The draft genome sequence of Protochlamydia sp. HS-T3 was 2,308,589 bp (2.3 Mb) with a G+C content of 38.74% and 950-fold genome coverage. The genome sequence contained 2,259 coding sequences, 40 tRNAs, and 2 ribosomal RNAs. Neighbor analysis using RAST revealed that the closest neighbor of Protochlamydia HS-T3 was Protochlamydia amoebophila UWE25 (NC_005861.1) (6), which is unable to grow in cells derived from higher forms of life, such as insects (13). Similarly to Protochlamydia UWE25, Protochlamydia HS-T3 was found by KEGG analysis to possess central carbon metabolic pathways (e.g., the Embden-Meyerhof pathway, tricarboxylic acid cycle, pentose phosphate pathway, Entner-Doudoroff pathway, and fatty acid biosynthesis) and type III general secretion gene clusters but lacked the genes encoding the type IV secretion system. In addition, 1,139 hypothetical proteins and 8 unknown proteins were identified.

### Nucleotide sequence accession numbers.

The draft genome sequence of Protochlamydia HS-T3 has been deposited at DDBJ/EMBL/GenBank under the accession numbers BBPT01000001 through BBPT01000034 (34 entries). The version described in this paper is the first version.
